# An Early Rehabilitation Favors the Prognosis of Hypertensive Intracerebral Hemorrhage With Acute Disorders of Consciousness: A Retrospective Cohort Study With Propensity Score Matching

**DOI:** 10.1155/np/8144313

**Published:** 2025-04-24

**Authors:** Rao Xu, Yi Sun, Lin Zhao, Ying Wang, Danjing Yu, Yunxiang Chen, Liqing Bi, Zhiyan Shen, Xintong Zhang, Wei Yan, Xi Wang

**Affiliations:** ^1^Department of Rehabilitation Medicine, The Affiliated Suqian First People's Hospital of Nanjing Medical University, Suqian 223800, China; ^2^Department of Neurosurgery, The First Affiliated Hospital with Nanjing Medical University, Nanjing 210029, China; ^3^Department of Rehabilitation Medicine, The First Affiliated Hospital with Nanjing Medical University, Nanjing 210029, China

**Keywords:** acute disorders of consciousness, early rehabilitation, hypertensive intracerebral hemorrhage, outcome assessment

## Abstract

**Objective:** There currently lacks the prognosis assessment of hypertensive intracerebral hemorrhage (HICH) with acute disorders of consciousness (DoC) after early rehabilitation (ER). The present study aims to investigate the outcomes of consciousness and neurological and cognitive functions in HICH patients with acute DoC intervened with ER via a retrospective cohort study with propensity score matching (PSM).

**Methods:** A total of 265 eligible HICH patients with acute DoC admitted to the First Affiliated Hospital with Nanjing Medical University from January 2021 to December 2023 were retrospectively recruited. They were randomly divided into the ER group (*n* = 115) and the nonER group (*n* = 150) before PSM. After the PSM at a ratio of 1:1, 96 patients were allocated to each group. Baseline characteristics before and after PSM were compared between the ER group and the nonER group. Outcome measures included the duration of mechanical ventilation, and proportions of participants with an emergence to a conscious state (eMCS), 0–3 points of the modified Rankin Scale (mRS), and cognitive impairment.

**Results:** Baseline characteristics were comparable between the ER group and the nonER group after PSM (*p* ≥ 0.05). An ER significantly shortened the duration of mechanical ventilation (9 days vs. 10 days, *p*=0.022). The neurological prognosis at 3 months of HICH combined with acute DoC was significantly improved by the ER, with a significantly higher proportion of participants grading 0–3 points of the mRS in the ER group than the nonER group (57.3% vs. 40.6%, *p*=0.021). Among 174 participants who restored consciousness at 3 months of onset, a significantly lower proportion of cognitive impairment was detected in the ER group than the nonER group (25.8% vs. 53.2%, *p*=0.002).

**Conclusion:** An ER shortens the duration of mechanical ventilation and improves the neurological prognosis in HICH patients with acute DoC. Although the outcome of consciousness is unable to be improved, an ER does reduce the risk of residual cognitive dysfunction in HICH patients with acute DoC.

## 1. Introduction

Hypertensive intracerebral hemorrhage (HICH) is the most frequent type of hemorrhagic stroke (HS), leading to the highest incidence [1]. Impaired consciousness at post HICH is one of the serious clinical symptoms. With the great strides made in medical techniques, the mortality of HICH patients with acute disorders of consciousness (DoC) has declined. Nevertheless, survived HICH patients with acute DoC have greatly suffered from long-term impaired consciousness and cognitive dysfunction, resulting in a very low quality of life and a heavy burden on the family and society [2]. An early rehabilitation (ER) is a potential treatment to improve the neurological prognosis of HICH. Previous evidence has shown that an ER significantly lowers the incidence of the shoulder-hand syndrome and depression, and elevates the survival of HICH by enhancing the capacities of daily activities and motor function in HS patients [3–5]. An ER provides survival benefits to critically ill patients in the intensive care unit (ICU) that shortens the length of stay in the ICU and the duration of mechanical ventilation [6, 7]. Currently, the potential influence of an ER on the prognosis of HICH patients with acute DoC has been rarely concerned. Given that patients with acute DoC are unable to actively participate in rehabilitation therapy, early bedside rehabilitation for these patients has historically been underemphasized. In recent years, our center has implemented early passive rehabilitation training for patients with HICH combined with acute DoC. Nevertheless, the extent to which ER treatment can improve the prognosis of HICH patients with acute DoC remains to be elucidated. In the present retrospective cohort study, we comprehensively assessed the effects of an ER on the prognosis of consciousness, neurological function, and cognitive function in HICH patients with acute DoC with propensity score matching (PSM). The results of this study may offer a scientific foundation for the ER of patients with acute DoC and possess potential for broader clinical implementation.

## 2. Materials and Methods

### 2.1. Participants

This was a retrospective, single-center cohort study initially involving 877 intracerebral hemorrhage (ICH) patients in the Neurosurgical ICU, the First Affiliated Hospital with Nanjing Medical University, from January 2021 to December 2023. Informed consent was provided by all participants. The present study was approved by the Ethics Committee of the First Affiliated Hospital with Nanjing Medical University (no. 2023-SR-378).

Inclusion criteria: (ⅰ) adults at 18 years of age and above, (ⅱ) combined with acute DoC with the onset Glasgow Coma Scale (GCS) score of 12 points and below, (ⅲ) admitted in ICU within 24 h of the first-onset HICH confirmed by computer tomography (CT), and (ⅳ) history of hypertension. Exclusion criteria: (ⅰ) ICU stay of less than 1 week; (ⅱ) traumatic cerebral hemorrhage; (ⅲ) combined with malignancies, limb or vertebral fractures, structural blood vessel abnormalities, coagulation or hematological disorders, and mental illnesses; (ⅳ) unstable vital signs after 3 days of onset; (ⅴ) treatment abandonment; and (ⅵ) death during hospitalization.

Finally, a total of 265 eligible participants were retrospectively recruited.

### 2.2. Data Collection

The following data were collected: age, sex, the minimum GCS score on the day of onset, preoperative cerebral herniation, history of diabetes mellitus, site of HICH, intraventricular hemorrhage, volume of HICH, preoperative midline shift (MLS) of the brain, postoperative secondary bleeding, epileptic seizures during hospitalization, secondary cerebral infarction, and the date of starting and weaning from mechanical ventilation.

### 2.3. Definitions

i. The volume of HICH was measured using the Picture Archiving and Communication System (PACS).
ii. On the axial plane, the MLS at the interventricular foramen was measured using the formula MLS = *A*/2−*B*, where *A* denotes the width of the intracranial space, and *B* is the distance from the septum pellucidum to the tabula interna [8].
iii. The Coma Recovery Scale-Revised (CRS-R) was used to assess consciousness. It is a 23-point scale composed of six subscales of the auditory function (0–4 points), visuoperception (0–5 points), motor functions (0–6 points), receptive and expressive language (0–3 points), communication ability (0–2 points), and arousal level (0–3 points). An emergence to a conscious state (eMCS) is determined by the grading of functional object use in the subscale of motor functions (6 points) or reliable yes or no responses in the communication subscale (2 points); otherwise, prolonged DoC (pDoC) are confirmed [9].
iv. The Montreal Cognitive Assessment (MoCA) was used to assess cognitive functioning. Cognitive impairment was defined as the MoCA score of 25 points or below [10]. The utilization of the MoCA score in this research has obtained the permission of MoCA Test Inc.
v. The modified Rankin Scale (mRS) was used to measure the neurological prognosis, with 0–3 points indicating a mild-to-moderate disability.

### 2.4. ER Procedures

A routine clinical management of HICH was given to all participants, including the evacuation of intracerebral hematomas, nutritional supplementation, diuresis, dehydration, blood pressure management, and supportive nursing care. A passive ER was performed within 3 days of admitting in ICU, including the normal limb position, passive movement of joints, breathing exercises, and sensory stimulation. Normal limb position was sustained continuously, other ER projects were carried out once daily.

ER protocol: (ⅰ) normal limb position. In the healthy lateral decubitus position, the affected upper limb was extended forward with the shoulder joint flexed at approximately 90°; the affected lower limb was positioned with the knee flexed and supported on a soft pillow, while the healthy lower limb remained extended. In the affected lateral decubitus position, the affected shoulder joint was maintained in a flexed position, the elbow joint was fully extended, and the fingers were held in a straightened position; the affected lower limb was kept extended with the knee joint slightly flexed, while the healthy lower limb was positioned with the knee joint moderately flexed. In the supine position, a pillow was placed beneath the patient's head and another beneath the shoulder joint on the affected side to maintain the anterior protrusion of the scapula; a sponge pad was positioned under the affected hip to ensure that the patient's pelvis was maintained in an anteriorly tilted position. The patients' positions were changed every 2 h. (ⅱ) Passive movement of joints for 10 min. The passive range of motion of the wrist, shoulder, hip, knee, and ankle joints was maintained through gentle traction and controlled rotational exercises. (ⅲ) Breathing exercise for 5 min. The therapist positioned themselves at the patient's head, with both hands contacting the underside of the costal cartilages of the seventh to tenth ribs to conduct manual diaphragm release treatment. During the inhalation phase, the contact points were gently pulled in a cephalad direction and slightly laterally while following the upward movement of the ribs; during the exhalation phase, the therapist applied deeper pressure towards the inner margin of the ribs and maintained resistance. (ⅳ) Auditory stimulation for 10 min. The patient's favorite music before the onset of HICH was chosen. If not possible to obtain, classical music was employed. (ⅴ) Visual stimulation for 5 min. A picture of the family member with whom the patient had the closest relationship was presented to the patient. The picture was cyclically shifted at a position 30 cm in front of the patient's eyes, first slowly moving 45° to the left and right along the vertical midline and then slowly moving 45° above and below along the horizontal midline. (ⅵ) Tactile stimulation for 5 min. Firm pressure was exerted with the fingertips from the patient's shoulder to the wrist.

### 2.5. Follow-Up

A 3-month follow-up was conducted after the onset of HICH. The consciousness status and neurological prognosis were assessed at 3 months of HICH with acute DoC. Cognition was additionally assessed in those with eMCS.

### 2.6. Statistical Analysis

Statistical analysis was performed with SPSS 23.0 (SPSS Inc., Armonk, NY, USA), the R package (version 3.1.3), and PSM plug-ins (version 3.0.4). The Shapiro-Wilk test for normality revealed that all continuous variables in the present study exhibited non-normal distributions and were therefore expressed as the median and interquartile boundary values (P25, P75). They were compared by the Mann-Whitney *U* test between groups. Categorical variables were expressed as frequencies and percentages, and compared using the chi-square test or Fisher's exact test. *p* < 0.05 was considered statistically significant.

The 1:1 ratio PSM was applied to eliminate potential bias and minimize the effects of 12 confounding factors (age, sex, the minimum GCS score on the day of onset, preoperative cerebral herniation, history of diabetes mellitus, site of HICH, intraventricular hemorrhage, volume of HICH, preoperative MLS, postoperative secondary bleeding, epileptic seizures during hospitalization, and secondary cerebral infarction) when estimating outcomes between the ER group and nonER group. Nearest-neighbor matching was conducted using the estimated propensity scores of patients. A match was established when a patient in the nonER group had an estimated score within 0.2 standard deviations (caliper width) of a patient in the ER group. The menu options were configured as follows: sampling without replacement, maximized execution performance, and randomization of case order during the matching process.

## 3. Results

### 3.1. Baseline Characteristics

**Figure 1:**
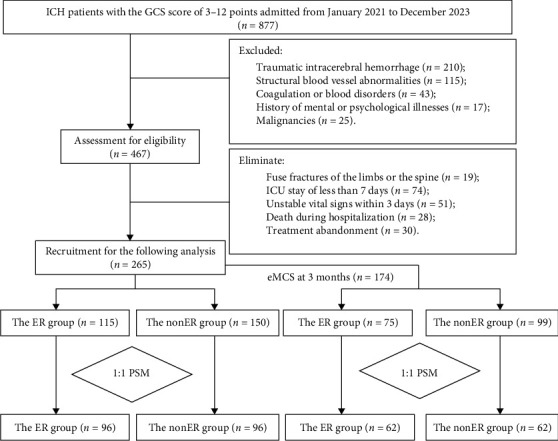
A flow chart of participant allocation. A total of 877 participants were initially recruited, after exclusion process, a total of 265 participants were finally included. After adjusting for intergroup differences using propensity score matching (PSM), 96 patients were included in both the ER group and the nonER group. Among 265 patients, 174 cases reached the eMCS state within 3 months of disease onset. These patients were classified into the ER group and the nonER group through PSM, with 62 patients in each group.

A total of 877 participants were initially recruited, and 265 were finally included in the present study, including 115 in the ER group and 150 in the nonER group (Figure 1). Baseline characteristics of them before and after PSM are listed in Table 1. Before PSM, the GCS score (*p*=0.013), site of HICH (*p*=0.039), epileptic seizures during hospitalization (*p*=0.042), and secondary cerebral infarction (*p*=0.042) were poorly balanced between the ER group and the nonER group. After PSM at a ratio of 1:1, 96 participants were included in either group, and no significant differences in the baseline characteristics were detected between groups (*p* ≥ 0.05).

### 3.2. An ER Favors the Weaning off Mechanical Ventilation, Neurological Improvement, and Cognitive Function in HICH Patients With Acute DoC

The duration of mechanical ventilation in the ER group was significantly shorter than the nonER group (9 days vs. 10 days, *p*=0.022). An ER was unable to significantly improve the consciousness in HICH patients with acute DoC at 3 months of onset, with a comparable proportion of eMCS between the ER group and the nonER group (68.8% vs. 66.7%, *p*=0.758). Notably, the neurological prognosis at 3 months of HICH combined with acute DoC was significantly improved by the ER. The proportion of participants grading 0–3 points of the mRS at 3 months was significantly higher in the ER group than that of the nonER group (57.3% vs. 40.6%, *p*=0.021) (Table 2).

We further analyzed the benefits of an ER on the cognitive function at 3 months in HICH patients with acute DoC. Among 265 participants, 174 restored consciousness at 3 months of onset, including 75 in the ER group and 99 in the nonER group (Table 3). Before PSM, the GCS score (*p*=0.009) and secondary cerebral infarction (*p*=0.040) were poorly balanced in consciousness awakening patients between the ER group and the nonER group. No significant differences in the baseline characteristics were identified after PSM (*p* ≥ 0.05). Cognitive functioning was assessed in 174 participants who restored consciousness at 3 months of onset by the MoCA scale. A significantly lower proportion of cognitive impairment was detected in the ER group than the nonER group (25.8% vs. 53.2%, *p*=0.002) (Table 4).

## 4. Discussion

Stroke is the leading cause of disability worldwide, leaving a series of sequelae [11]. More seriously, stroke survivors face a poor quality of life due to the dependance on daily activities [12]. Neuroplasticity at poststroke allows remarkable restorative abilities of the brain, the impaired central nervous system, however, still lacks effective medications and approaches to be improved [13]. Existing evidence has highlighted the role of enhancing the spontaneous nerve repair process and improving nervous system plasticity in improving the prognosis of stroke. Rehabilitation exercises are a promising, noninvasive, economically friendly way to accelerate the recovery of impaired neurological function at poststroke [14, 15]. Skilled reaching training is found to stimulate astrocytic plasticity and recovery following an ICH in rats [16]. There exists a time window in the plasticity stage of the central nervous system [17], and the advance of an ER prior to the ICU treatment has become the latest spotlight of research. Previous studies have analyzed the efficacy and safety of an ER on patients with ICH [5, 18]. Targeted at the specified population of HICH patients with acute DoC, the present study assessed the role of a passive ER in improving the prognosis.

Our data revealed that an ER significantly shortened the duration of mechanical ventilation in HICH patients with acute DoC. Pang et al. [19] consistently reported a shorter duration of mechanical ventilation in ICU patients with cerebral hemorrhage or traumatic brain injury who received ER therapy. A reduction in the duration of mechanical ventilation not only decreases ICU length of stay and associated costs but also lowers the incidence of ventilator-associated pneumonia, thereby improving overall patient outcomes. Critically ill patients in the ICU usually lose spontaneous movement or are immobilized, leading to a decreased diaphragm strength and impaired discharge of secretions, both of which can induce pneumonia and prolong mechanical ventilation [20]. An ER performed in our center was composed of the normal limb position, passive movement of joints, breathing exercises, and sensory stimulation. Breathing exercise is conducive to promote the respiratory muscle activities and facilitate the clearance of airway secretions, thereby shortening the duration of mechanical ventilation [21, 22]. Upper limb exercises are capable of partially controlling the contraction of the diaphragm, suggesting that a passive activity training of limb joints may also promote the recovery of diaphragm function [23].

By measuring the CRS-R score at 3 months after onset of HICH combined with acute DoC, an ER was found to be unable to improve the outcome of consciousness. In cohorts of traumatic brain injury combined with DoC, an early sensory stimulation greatly promotes the recovery of consciousness [24, 25]. Inconsistently, sensory stimulation in the ER protocol did not improve the state of consciousness in HICH patients with acute DoC, which may be attributed to the differences in mechanisms underlying the HICH-induced neurological damage and the implementers responsible for the ER. All ER procedures in our study were performed by experienced rehabilitation therapists. However, a family-centered sensory stimulation allows a superior outcome of consciousness awakening in comatose patients with traumatic brain injuries than a sensory stimulation performed by nurses or rehabilitation therapists [26]. Moreover, the duration of ER is a critical factor influencing the prognosis of consciousness. It is found that a minimum of 2 weeks of sensory stimulation is required to influence the level of consciousness [27].

The effect of ER on improving neurological function may be greatly attributed to sensory stimulation and passive activity training of limb joints. Sensory stimulation effectively improves neurological and motor functions in patients with acute stroke [28]. In our study, a significantly milder neurological disability was found in the ER group than the nonER group, indicating that an ER favored the recovery of motor function and self-care ability in HICH patients with acute DoC. Passive motion is beneficial to maintain the shoulder joint stability following an acute stroke [29]. Functional magnetic resonance imaging (fMRI) suggested an elevated activation of the ipsilateral and contralateral cortex in stroke patients managed by the passive motion [30].

We conducted the MoCA assessment on HICH patients with eMCS at 3 months post-onset and found that ER reduced the risk of cognitive impairment after consciousness recovery. Given that cognitive function can only be assessed in awake patients, this approach may introduce selection bias. Nonetheless, it still partially reflects the positive impact of ER on cognitive outcomes. An ER significantly reduced the risk of cognitive impairment in HICH patients after the awakening of consciousness, probably due to the multisensory stimulation. We have applied auditory, visual, and tactile stimulations to DoC patients in our center. Davis and Gimenez [31] reported the role of an auditory stimulation in promoting cognitive function recovery after traumatic brain injuries. In neurodegenerative disease patients with the major manifestation of cognitive impairment, an active auditory stimulation also provides benefits to the improvement of cognition [32]. Auditory stimulation improves learning and memory functions mainly through activating neurogenesis in the mammalian hippocampus and enhancing neural connections [33]. In a traumatic brain injury model in rats, a combination of visual and tactile stimulations effectively stimulates the recovery of cognitive function [34]. In the future, an individualized ER via multisensory stimulation is expected to further improve the neurological prognosis of HICH combined with acute DoC.

Our study demonstrated the positive impact of ER on the prognosis of patients with HICH combined with acute DoC. Nevertheless, this study is subject to certain limitations. The single-center retrospective design may introduce selection bias in patient enrollment. In future investigations, we aim to conduct a multicenter prospective study to further validate the therapeutic efficacy of ER. Moreover, while our center has implemented diverse passive rehabilitation interventions, the influence of the duration and frequency of each treatment modality on patient outcomes remains to be elucidated in subsequent studies. Beyond HICH, other neurological injury conditions, such as traumatic brain injury and aneurysmal subarachnoid hemorrhage, are also frequently associated with acute DoC. Investigating the therapeutic potential of ER for these conditions holds substantial clinical significance.

## 5. Conclusion

An ER shortens the duration of mechanical ventilation and improves the neurological and cognitive prognosis in HICH patients with acute DoC. It is a feasible approach to be promoted in the clinical management of HICH.

## Figures and Tables

**Table 1:** Baseline characteristics before and after PSM.

Variables	Before PSM (*n* = 265)			After PSM (*n* = 192)		
	ER group (*n* = 115)	NonER group (*n* = 150)	*p*-value	ER group (*n* = 96)	NonER group (*n* = 96)	*p*-value
Male (*n*, %)	79 (68.7%)	114 (76.0%)	0.185	70 (72.9%)	70 (72.9%)	1.000
Age (years)	58 (48.5,66.5)	58 (50,67)	0.251	58 (48.3,67)	56 (49,66.8)	0.860
GCS score (points)	8 (5,9.5)	8 (6,10)	0.013	8 (6,10)	8 (6,10)	0.754
Cerebral hernia (*n*, %)	17 (14.8%)	18 (12.0%)	0.507	14 (14.6%)	10 (10.4%)	0.383
History of diabetes (*n*, %)	28 (24.3%)	26 (17.3%)	0.160	18 (18.8%)	18 (18.8%)	1.000
Site of HICH (*n*, %)	—	—	0.039	—	—	0.074
Basal ganglia	76 (66.1%)	86 (57.3%)	—	68 (70.8%)	61 (63.5%)	—
Thalamus	4 (3.5%)	9 (6.0%)	—	3 (3.1%)	8 (8.3%)	—
Cerebral lobes	19 (16.5%)	45 (30.0%)	—	15 (15.6%)	24 (25.0%)	—
Brainstem	7 (6.1%)	4 (2.7%)	—	4 (4.2%)	1 (1.0%)	—
Cerebellum	9 (7.8%)	6 (4.0%)	—	6 (6.3%)	2 (2.1%)	—
Volume of HICH (mm^3^)	29 (19,40)	25 (15,40)	0.541	30 (20,40.8)	27.5 (15,40)	0.469
Intraventricular hemorrhage (*n*, %)	50 (43.5%)	63 (42.0%)	0.809	42 (43.8%)	37 (38.5%)	0.463
MLS (mm)	4.5 (0,8)	5.0 (0,8)	0.620	5 (0.5,8)	4 (2,7.8)	0.536
Secondary bleeding (*n*, %)	6 (5.2%)	3 (2.0%)	0.183	4 (4.2%)	3 (3.1%)	1.000
Epileptic seizures (*n*, %)	18 (15.7%)	39 (26.0%)	0.042	18 (18.8%)	21 (21.9%)	0.590
Secondary cerebral infarction (*n*, %)	31 (27.0%)	25 (16.7%)	0.042	23 (24.0%)	21 (21.9%)	0.731

Abbreviations: ER, early rehabilitation; GCS, the Glasgow Coma Scale; HICH, hypertensive intracerebral hemorrhage; MLS, midline shift; PSM, propensity score matching.

**Table 2:** Neurological prognosis of HICH.

Outcomes	ER group (*n* = 96)	NonER group (*n* = 96)	*p*-Value
Duration of MV (days)	9 (8,12)	10 (8,15)	0.022
eMCS at 3 months (n, %)	66 (68.8%)	64 (66.7%)	0.758
0–3 points of the mRS (n, %)	55 (57.3%)	39 (40.6%)	0.021

Abbreviations: eMCS, emergence to a conscious state; ER, early rehabilitation; HICH, hypertensive intracerebral hemorrhage; mRS, modified Rankin Scale; MV, mechanical ventilation.

**Table 3:** Baseline characteristics of participants who restored the consciousness at 3 months of onset.

Variables	Before PSM (*n* = 174)			After PSM (*n* = 124)		
	ER group (*n* = 75)	NonER group (*n* = 99)	*p*-value	ER group (*n* = 62)	NonER group (*n* = 62)	*p*-value
Male (*n*, %)	51 (68.0%)	79 (79.8%)	0.076	48 (77.4%)	47 (75.8%)	0.832
Age (years)	56 (48,63)	57 (50,67)	0.109	55.5 (42.8,62.3)	53.5 (47.5,61.3)	0.745
GCS score (points)	8 (6,10)	9 (7,11)	0.009	8 (6.8,10)	8.5 (7,10)	0.756
Cerebral hernia (*n*, %)	9 (12.0%)	8 (8.1%)	0.389	7 (11.3%)	7 (11.3%)	1.000
History of diabetes (*n*, %)	13 (17.3%)	16 (16.2%)	0.837	11 (17.7%)	13 (21.0%)	0.649
Site of HICH (*n*, %)	—	—	0.065	—	—	0.250
Basal ganglia	51 (68.0%)	54 (54.5%)	—	44 (71.0%)	38 (61.3%)	—
Thalamus	1 (1.3%)	5 (5.1%)	—	1 (1.6%)	3 (4.8%)	—
Cerebral lobes	13 (17.3%)	33 (33.3%)	—	9 (14.5%)	17 (27.4%)	—
Brainstem	3 (4.0%)	2 (2.0%)	—	3 (4.8%)	2 (3.2%)	—
Cerebellum	7 (9.3%)	5 (5.1%)	—	5 (8.1%)	2 (3.2%)	—
Volume of HICH (mm^3^)	25 (17,40)	20 (15,33)	0.131	25 (15,33.5)	20 (14.5,36)	0.382
Intraventricular hemorrhage (*n*, %)	31 (41.3%)	36 (36.4%)	0.505	24 (38.7%)	24 (38.7%)	1.000
MLS (mm)	4 (0,8)	3 (0,5)	0.167	3 (0,7)	3 (0,5)	0.470
Secondary bleeding (*n*, %)	2 (2.7%)	2 (2.0%)	1.000	2 (3.2%)	0 (0.0%)	0.496
Epileptic seizures (*n*, %)	13 (17.3%)	26 (26.3%)	0.162	12 (19.4%)	13 (21.0%)	0.823
Secondary cerebral infarction (*n*, %)	18 (24.0%)	12 (12.1%)	0.040	12 (19.4%)	8 (12.9%)	0.329

Abbreviations: ER, early rehabilitation; GCS, the Glasgow Coma Scale; HICH, hypertensive intracerebral hemorrhage; MLS, midline shift; PSM, propensity score matching.

**Table 4:** Cognitive prognosis of HICH patients who restored the consciousness at 3 months of onset.

Outcomes	ER group (*n* = 62)	NonER group (*n* = 62)	*p*-Value
Cognitive impairment at 3 months (*n*, %)	16 (25.8%)	33 (53.2%)	0.002

Abreviations: ER, early rehabilitation; HICH, hypertensive intracerebral hemorrhage.

## Data Availability

The clinical and imaging dataset analyses during the current study are available from the corresponding author on reasonable request.
